# Workplace-Based Exercise Intervention Improves Work Ability in Office Workers: A Cluster Randomised Controlled Trial

**DOI:** 10.3390/ijerph16152633

**Published:** 2019-07-24

**Authors:** Joshua Zheng Rui Ting, Xiaoqi Chen, Venerina Johnston

**Affiliations:** 1 School of Health and Rehabilitation Sciences, The University of Queensland, Brisbane 4072, Australia; 2 RECOVER Injury Research Centre, The University of Queensland, Brisbane 4006, Australia

**Keywords:** work ability, neck/shoulder strengthening exercises, office workers, neck pain

## Abstract

Neck pain is a burden to employers and employees amenable to improvement with neck/shoulder strengthening exercises. However, the benefits of such interventions on office workers’ work ability remains unknown. This study evaluated the effects of a 12-week combined ergonomics and neck/shoulder strengthening exercise intervention (EET, *n* = 177, mean age 41.7 years, 26% female), versus a 12-week combined ergonomics and health promotion intervention (EHP, *n* = 173, mean age 43 years, 29% female) on work ability among office workers. Work ability was measured by a single question. Differences in the work ability score were analyzed using the intention-to-treat (ITT) and per-protocol (i.e., adherence ≥70%) analyses for between- and within-group differences at baseline, 12 weeks, and 12 months. A sub-group analysis was performed for neck cases, defined as reporting neck pain as ≥3 (out of 10). No significant between-group differences for work ability were observed in the general population, and subgroup of neck cases. A significant group-by-time interaction effect at 12 weeks and the trend for significance at 12 months favored the EET group in the per-protocol analysis of the neck cases. EET was effective in increasing work ability post-intervention and potentially, in the long-term, in symptomatic participants with ≥70% adherence to the intervention. However, EET was not superior to EHP.

## 1. Introduction

Musculoskeletal pain is a threat to productivity. In particular, neck pain places a significant burden on both the individual and employer due to the costs associated with treatment, reduced productivity, and work absenteeism [1,2,3,4]. As non-specific neck pain is a common and episodic musculoskeletal disorder in office workers [2], it is not surprising that many interventions to address this problem have been developed and tested.

Interventions tested with office workers tend to focus on the reduction of neck symptoms through exercise or changes to the ergonomic work environment [5,6,7]. The evidence from several systematic reviews, however, consistently demonstrates neck/shoulder strengthening exercises to be of greater benefit in reducing musculoskeletal pain in office workers than ergonomic interventions [7,8,9]. Despite this evidence, the impact of neck and shoulder exercise interventions on work ability is limited.

Our research group recently demonstrated that health-related productivity loss can be positively impacted with a workplace-based combined intervention of ergonomics and neck/shoulder exercises in office workers [10]. Thus, it is possible that such a workplace-based intervention will also result in improvements in work ability. The concept of work ability was introduced to define a person’s capacity to meet the demands of their work considering their health, personal resources, and work environment [11]. Poor work ability is associated with early retirement, work disability, and productivity loss due to absenteeism [12,13] and long term sickness absence [14]. A limited number of studies have investigated methods to improve work ability, mostly in occupational groups performing physically demanding roles (e.g., cleaners and laundry workers [15,16,17,18]) due to the perception that their work ability is impaired to a greater degree compared to those performing mostly mentally demanding work (e.g., office workers). Some interventions tested included cognitive behavioral therapy [18], generic aerobic exercise (such as dancing, walking or cycling [16,18,19]), region specific exercise interventions delivered only to those with pain [17], or a combination of muscle strengthening, stretching, and cardiovascular exercise [15]. For office workers, interventions tested to impact work ability have been ergonomic [20] or exercise [21]. Based on the evidence to date, it appears that interventions that are multimodal (i.e., contain more than one type of intervention), including strengthening exercises targeting a specific painful region, are more likely to be more efficacious in improving work ability in office workers.

This study is nested within a recently completed 12-week cluster randomized trial that investigated the impact of a combined ergonomics and neck/shoulder strengthening exercise (EET) intervention compared to a combined ergonomics and health promotion (EHP) intervention in a sample of Australian office workers. This is a secondary analysis of a subsample of office workers who provided data on work ability at baseline, immediately after the intervention, and 12 months after commencement. It was hypothesized that the EET intervention would be more efficacious for improving work ability in office workers compared to EHP, and that this effect would be stronger for those with neck pain at baseline.

## 2. Materials and Methods

A prospective parallel two-arm cluster-randomized trial (ACTRN12612001154897) was undertaken from May 2013 to July 2016. The study was conducted in accordance with the Declaration of Helsinki, and the protocol was approved by the Medical Research Ethics Committee of The University of Queensland (#2012001318). The protocol has been published [22] and the CONSORT extension for cluster trials adopted for reporting [23] (Supplementary material Table S1).

### 2.1. Participants

The participants of this study were recruited from 14 private and government organizations in Brisbane, Queensland. The inclusion criteria were any office worker over 18 years working more than 30 hours per week. Participants were ineligible if they were pregnant, had a previous neck injury or trauma or inflammatory condition, a history of neck surgery, or were unable to exercise due to any medical conditions. Only 366 of the 763 participants recruited into the study received the question related to work ability and constitute the sample for this study (Table 1).

### 2.2. Procedure

All participants were allocated to clusters of five to eight individuals according to their office building, floor, or department. This approach was undertaken to minimize contamination of interventions and enhance compliance with the intervention. The clusters were then randomly allocated to either EET or EHP by a blinded statistician using computer-generated block randomization (in blocks of four). Participants were encouraged to continue with their usual physical activity while participating in the research. The ergonomic, exercise, and health education interventions were conducted during work time onsite. This was to minimize business disruption and enhance participation and adherence.

Prior to the commencement of their respective interventions, both groups were provided with one-on-one, best practice ergonomic assessment using a 37 item checklist across seven sub-categories (chair, desk, monitor, keyboard, mouse, telephone, and the physical environment (e.g., lighting, temperature, and noise) based on the local government guidelines [24]. The assessment and intervention were prescribed by a qualified health professional, consisting of a one-hour initial assessment with up to one follow-up session. Adjustments may have been minor, such as advice or education (e.g., take frequent breaks), physical changes to their workstation (e.g., raising the height of their chair), or new equipment (e.g., a headset).

**Figure 1:**
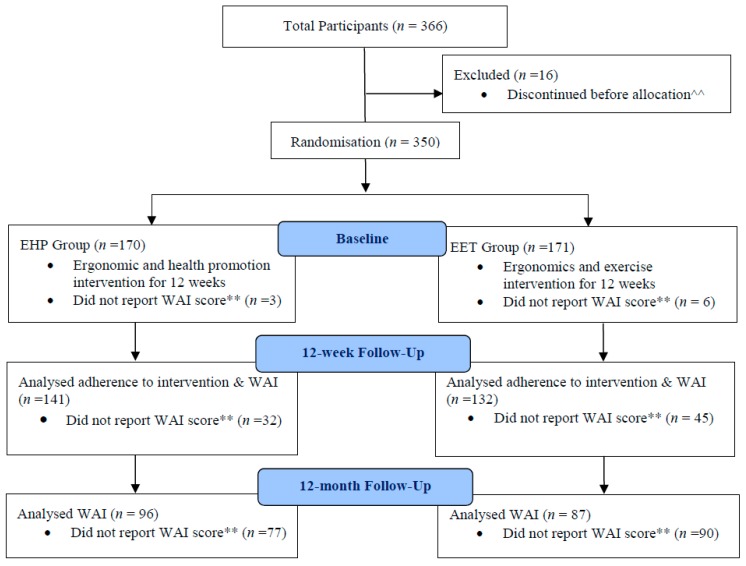
Flow of participants through the study. WA = Work Ability; **^^** 16 Participants discontinued before allocation: 13 excluded during assessment, 2 due to excessive work demands, 1 due to other reasons; ** Reasons for failure to report work ability scores (1) Participant discontinued due to pregnancy, unrelated illness or injury, change of employer, excessive work demands, or other unstated reasons (2) Participant did not complete response due to: absence for data collection, incomplete response if participants cease surveys part way through or if the online survey portal failed to force the response. EHP = ergonomics and health promotion intervention; EET = ergonomics and neck/shoulder strengthening exercise intervention.

Participants were allocated to clusters of 5–8 participants in the EET group and received progressive exercises three times per week for 12 weeks, with each session lasting no more than 20 minutes conducted in one of the rooms at the workplace. The exercises consisted of postural facilitation, shoulder, and neck exercises (seven exercises in total). The postural facilitation exercise was emphasized in the first 2 weeks. The upper neck flexion exercise were included as a warm up for each session, and the five main exercises were performed in cycles of three exercises/session (i.e., neck flexion, neck extension, front and side arm raise to 90°, and reverse flies) (further details are available in [22]). The five exercises were performed with weights or resistance bands and progressed throughout the intervention period using the principles of periodization and progressive overload. One of the three sessions was supervised by a physiotherapist who progressed the exercises as required. To ensure the correct completion of the exercises, participants were provided with a hard copy diary, which included an explanation of each exercise with accompanying photos. The diary also specified the exercise parameters (sets and repetitions) and which exercise was to be performed at each session for each week. Participants recorded exercises completed at each session in their diary. The EHP group was provided with a one hour health promotion session weekly for 12 weeks, which was facilitated by a different health professional. The weekly health promotion topics included healthy dieting, losing weight, discontinuance of smoking, relaxation, and managing stress. Further details regarding the interventions are published elsewhere [22]. Figure 1 displays the flow of participants through the study.

### 2.3. Primary Outcome

Work ability was measured by a single question, which has been validated to be accurate in predicting work ability compared with the original 7 item Work Ability Index [25]. This question required participants to rate their current work ability, compared to their work ability at its best, on an 11 point scale (from 0 to 10). Participants reported their work ability at three time points (baseline, post-intervention at 12 weeks and follow-up at 12 months). This data and all independent variables were collected via an online survey with the baseline data collected prior to randomization.

### 2.4. Independent Variables

Several individual and work-related factors potentially impacting work ability were assessed. Individual variables evaluated included the severity of neck pain (0–9 scale for pain in the last 7 days), physical activity using the International Physical Activity Questionnaire ((IPAQ), coded as Inactive/Minimally Active/Active) [26], and psychological distress measured with the Kessler 6 (K6) scale [27] (scores range from 0–15). Data on participant demographics (age, gender, BMI (kg/m^2^), education level (three levels), and existing total comorbidities were also collected and entered as covariates. Participants were asked to report their total comorbidities from a list of 15 common health conditions, such as cancer, asthma, osteoarthritis, and depression. Work-related factors evaluated at baseline included job satisfaction measured by a single question on a 7 point scale [28], occupational category (three categories), work industry (government/private), the duration of computer usage (</> 6 h per day), and total ergonomic score (assessed on a checklist with scores ranging from 0–38). Information relating to individuals who received workers’ compensation, or those who sought help from a healthcare professional (Yes/No) for their neck/shoulder symptoms in the last 12 months, were also obtained and evaluated at baseline.

Adherence during the 12-week intervention was recorded by the facilitators in the EET and EHP groups and analyzed as a total adherence percentage score. Adherence after the 12-week intervention was monitored but not reported here.

### 2.5. Data Analysis

The data collected were analyzed according to the intention-to-treat (ITT) and per-protocol principles for all participants. Multiple imputation was performed to impute missing data for individual variables (e.g., age and gender). A comparison of results (with and without imputation) showed no significant differences. The sub-sample analysis of the neck cases only, was also performed using ITT and per-protocol analysis. Cases were defined as those who reported neck pain of three or more on the 10-point scale at baseline [29]. The per-protocol analysis included participants with adherence of ≥70% during the 12-week intervention [30]. Descriptive statistics for between-group differences in the baseline characteristics were performed using t-test for continuous variables and chi-square test for categorical variables. Between-group and within-group changes in work ability were analyzed using the Multilevel Mixed Effects Linear Model with group, time, and group-by-time interactions as the fixed-effects independent variables. The analyses were controlled for age, gender, baseline ergonomic score, whether care was sought from a health professional, and BMI. Organization and cluster were entered into the analyses as random factors.

All data analyses were performed using STATA/IC (Statistical Software for Data Science) version 15 (Stata Corporation, College Station, TX, USA), adopting a statistical significance of *p* < 0.05.

## 3. Results

According to the ITT and per-protocol analyses, 350 participants comprised the final sample in this study, as 16 participants discontinued before group allocation (Figure 1). There were no significant between-group differences in any baseline measures. However, the mean total ergonomic score was statistically greater in the EET group (*p* < 0.05), albeit only by a mean of 0.86 (2%) points, and there was a greater proportion of participants who did not seek health professional help in both the EET and EHP groups (*p* < 0.05) (Table 1). For all participants, the mean (SD) baseline work ability score for the EET and EHP groups were 8.43 (1.27) and 8.45 (1.25) for the ITT analysis, and similarly in the per-protocol analysis, at 8.45 (1.23) and 8.52 (1.25), respectively. For the sub-sample of neck cases, the mean (SD) baseline work ability score for the EET and EHP groups was 8.31 (1.18) and 8.38 (1.27) for the ITT analysis, and 8.16 (1.17) and 8.42 (1.35) for the per-protocol analysis, respectively.

Adherence during the intervention period ranged from 0 to 100%. The EET intervention had a mean adherence of 60%, while the EHP group had a mean adherence of 55%. For the neck cases, the mean adherence was 63% for EET (8% to 100%) and 54% for EHP (0 to 92%). A total of 116 participants demonstrated ≥70% adherence to the allocated 12 week intervention, with 52% and 48% participants from the EET and EHP groups, respectively.

For all participants, there were no between-group differences, and no group-by-time interaction effect in work ability at 12 weeks or 12 months in the ITT and per-protocol analyses (Table 2). There was also a lack of significant between-group and group-by-time interaction effect for the neck cases using ITT analysis. There was, however, a significant group-by-time interaction effect for the per-protocol analysis of neck cases at 12 weeks (*p* < 0.05), and a near significant finding at 12 months (*p* = 0.06) that favored the EET group. This was reflected by the 0.66 (6%) and 0.77 (7%) improvement in mean (SD) work ability score from 8.16 (1.17) at baseline, to 8.82 (0.95) at 12 weeks, and 8.93 (1.14) at 12 months.

### Adverse Events

There were two participants who experienced musculoskeletal symptoms during the EET intervention. The intervention physiotherapist followed these participants who both completed the intervention without further problems.

## 4. Discussion

This study found no significant between-group differences in work ability when participating in an EET intervention or an EHP intervention. However, those who had neck pain at baseline and had completed more than 70% of the EET intervention showed an improvement in work ability at 12 weeks, which appeared to be maintained for the next nine months. In other words, for office workers with neck pain, the EET intervention was superior to the EHP intervention after 12 weeks. These findings partly support our hypothesis that the EET intervention could be more efficacious for improving work ability in office workers, compared to the EHP intervention, and that this effect would be stronger in those with neck pain at baseline.

The parent study, from which this study was derived, was powered to detect a difference in productivity loss between the two groups [10]. However, work ability data were only obtained from half of the sample, which may have been insufficient to detect a change in work ability. This is unlikely as the magnitude of the change in workability of 6%–7% is consistent with previous studies of a smaller (work ability improved by 7% [16]) and similar (work ability improved 4% [21]) sample size. As reported in another study with office workers, Justesen et al. [21] found that office workers had a greater improvement in work ability if adherence was 70% or more. A new finding from this research is that this change was greater in those with neck pain at baseline.

The relatively high mean baseline level of work ability in the moderate/good range [25] may have resulted in a ceiling effect, thereby mitigating the potential for a greater positive change at 12 weeks and 12 months. Various studies have found that the work ability scale may be prone to a ceiling effect [31]. Two intervention studies showed that improvement was especially significant for participants with poor to moderate baseline work abilities [19,31].

The EET intervention targeted individual physical factors, while the EHP intervention addressed psychosocial and work-related factors. The latter factors include health beliefs, mental health issues, healthy diet, job contentment, work attitudes, conflict management and resilience, and maintaining a healthy lifestyle. It has been hypothesized that work ability in office workers is not solely dictated by changes in physical activity but also changes in psychosocial well-being [32]. Therefore, the lack of difference between groups could have resulted from each intervention targeting a different aspect of work ability, with the EET intervention targeting the physical aspect and the EHP intervention targeting the psychosocial and work-related aspect. Another workplace intervention study in Australian office workers found a similar baseline work ability score (8.4/10, [33]). In their study, an activity-based work environment was introduced for 4 weeks but resulted in a reduction in work ability rather than the expected increase.

Future studies should consider interventions that combine exercise with psychosocial and work-related factors, as well as the contemporary workplace environment, as these domains affect work ability [13,33]. Future research should also consider revising the work ability questionnaire to include specific psychosocial and work-related factors, as it currently focuses more on physical factors. This may provide a more sensitive measure of work ability in office workers, the nature of whose work is more mentally demanding than physically demanding.

### Strengths and Limitations

These findings can be generalized to all office workers for several reasons. This study was rigorously conducted with a large sample of workers recruited from 14 different organizations, representing both the private and public government workforce. The clustering of participants according to their building, floor, or department was designed to encourage compliance within work teams and minimize contamination bias. Another strength was the range of individual and work-related characteristics evaluated and included for potential confounding.

The limitations of this study were the amount of missing data and low adherence to the intervention. However, adherence to interventions is a common problem reported in similar intervention studies varying from 56% [21] to 81% [17]. In our study, steps were taken to enhance adherence, such as ensuring that the exercises were easy to perform without the need to change or shower, conducted during work hours without leaving the workplace, and required very little equipment. Other measures introduced promoted a group champion and featured regular reminders via email. However, these strategies target the individual and there is evidence to indicate that a more holistic approach that considers the psychosocial work environment and organizational structures is needed to positively impact health behaviours in the workplace [34]. Indeed, a recent qualitative study investigating the barriers to physical exercise in the workplace suggests that the internal organizational working culture is the main barrier to compliance [35].

## 5. Conclusions

The main finding of this study is that a workplace-based ergonomics and exercise intervention is not superior to ergonomics and health promotion intervention in improving work ability in the general office worker population. However, this intervention was more efficacious in office workers with neck pain at baseline in the short- and potentially the long-term when 70% or more of the exercise sessions were completed. Further improvements in work ability may be possible with interventions that target individual, psychosocial, and work environments.

## Figures and Tables

**Table 1:** Baseline characteristics.

Variables	All Participants (*n* = 350)		Neck Cases Sub-Sample (*n* = 97)	
	EHP Group (*n* = 173)	EET Group (*n* = 177)	EHP Group (*n* = 52)	EET Group (*n* = 45)
Age (years), mean (SD)	43.00 (9.48)	41.68 (10.83)	41.37 (8.41)	41.46 (10.88)
Gender, *n* (%)				
Male	70 (21)	81 (24)	18 (19)	8 (8)
Female	100 (29)	90 (26)	34 (35)	37 (38)
Body Mass Index (kg/m^2^), mean (SD)	26.46 (5.33)	26.34 (5.79)	25.99 (4.24)	25.51 (5.55)
Occupational Category ^†^, *n* (%)				
Category 1	36 (11)	39 (11)	9 (9)	7 (7)
Category 2	87 (26)	86 (25)	31 (32)	22 (23)
Category 3	47 (14)	46 (13)	12 (12)	16 (16)
Industry, *n* (%)				
Private Sector	61 (18)	62 (18)	19 (20)	18 (19)
Government Sector	109 (32)	109 (32)	33 (34)	27 (28)
Highest Level of Education, *n* (%)				
Primary to Year 12	33 (10)	28 (8)	6 (6)	6 (6)
University	122 (36)	119 (35)	42 (43)	31 (33)
Trade College	15 (4)	24 (7)	4 (4)	8 (8)
Computer Hours/Day, *n* (%)				
Less Than 6 Hours	32 (9)	36 (11)	9 (9)	8 (8)
More Than 6 Hours	138 (40)	135 (40)	43 (44)	37 (38)
Total comorbidities, mean (SD) (0–5)	0.69 (0.92)	0.54 (0.90)	0.65 (0.88)	0.69 (0.99)
Job Satisfaction, mean (SD) (1–7)	4.92 (1.09)	4.78 (1.21)	4.83 (1.18)	4.69 (1.20)
Psychological Distress, mean (SD) (0–15)	3.56 (2.92)	3.81 (3.17)	4.06 (3.12)	4.24 (3.36)
Total ergonomic score, mean (SD) (0–38)	**31.37 (3.39)**	**32.23 (2.82)**	30.83 (3.37)	32.02 (2.92)
Severity of Neck Pain in the last 7 days, mean (SD) (0–9)	1.77 (2.26)	1.50 (2.00)	4.73 (1.65)	4.44 (1.42)
IPAQ ^∆^, *n* (%)				
Category 1	63 (18)	61 (18)	18 (19)	19 (20)
Category 2	90 (26)	91 (27)	30 (31)	22 (23)
Category 3	17 (5)	19 (6)	4 (4)	4 (4)

^†^ = Category 1 (manager or senior official), Category 2 (professional, associate professional, technical, or others), Category 3 (administrative, secretarial, or personal services); ^∆^ = Category 1 (Inactive), Category 2 (Minimally Active), Category 3 (HEPA active); boldface = significant between-group differences at *p* < 0.05; SD = standard deviation.

**Table 2:** Differences in mean work ability scores between groups over time for ITT and Per-protocol analyses ^a^.

	**EHP vs. EET All Participants**						
	Work Ability *	**ITT**			**Per-Protocol**		
		**b**	**95% CI**	***p***	**b**	**95% CI**	***p***
Group		0.02	−0.25 to 0.29	0.89	0.05	−0.41 to 0.51	0.83
Time	12 weeks	−0.01	−0.22 to 0.20	0.93	−0.05	−0.41 to 0.30	0.77
	12 months	−0.08	−0.32 to 0.17	0.54	−0.09	−0.49 to 0.31	0.66
Group × Time	12 weeks	0.24	−0.06 to 0.55	0.12	0.24	−0.26 to 0.73	0.35
	12 months	0.11	−0.24 to 0.46	0.53	0.14	−0.40 to 0.68	0.61
	**EHP vs. EET in Neck Cases**						
	**Work Ability** *	**ITT**			**Per-Protocol**		
		**b**	**95% CI**	***p***	**b**	**95% CI**	***p***
Group		−0.16	−0.67 to 0.34	0.52	−0.53	−1.24 to 0.17	0.14
Time	12 weeks	−0.03	−0.43 to 0.37	0.88	−0.37	−1.02 to 0.28	0.27
	12 months	−0.17	−0.65 to 0.32	0.50	−0.46	−1.20 to 0.27	0.21
Group × Time	12 weeks	0.40	−0.21 to 1.01	0.20	1.11	0.14 to 2.08	0.03
	12 months	0.54	−0.17 to 1.25	0.14	1.02	−0.05 to 2.08	0.06

** Baseline Work ability was used as the reference. ^a^ Analyses were adjusted for age, gender, BMI, healthcare professional sought for neck/shoulder symptoms in the last 12 months and Total Ergonomic Score. b = beta coefficient; CI = Confidence Interval; Group × Time = Group-by-time interaction. boldface = significant between-group differences at *p* < 0.05.

## Supplementary Materials

The following are available online at https://www.mdpi.com/1660-4601/16/15/2633/s1, Table S1: CONSORT 2010 checklist of information to include when reporting a randomised trial.
